# The Relationship Between Immune Semaphorins and Myasthenia Gravis

**DOI:** 10.1002/brb3.71291

**Published:** 2026-02-27

**Authors:** Dilcan Kotan, Esen Çiçekli, Özlem Aydemir

**Affiliations:** ^1^ Neurology Department Sakarya University Faculty of Medicine Sakarya Turkey; ^2^ Neurology Department Sakarya University Education and Research Hospital Sakarya Turkey; ^3^ Clinical Microbiology Department Sakarya University Faculty of Medicine Sakarya Turkey

**Keywords:** myasthenia gravis, semaphorin 3A, semaphorin 3F, semaphorin 4A, semaphorin 4D, semaphorin 7A

## Abstract

**Objective:**

Myasthenia gravis (MG) is an autoimmune disease involving several immune mechanisms. Recently, semaphorins have emerged as potential diagnostic and prognostic biomarkers in autoimmune neurological and non‐neurological diseases. This study investigated the role of immune semaphorins, namely semaphorins 3A, 3F, 4A, 4D, and 7A, in the diagnosis and prognosis of MG and their potential as biomarkers.

**Methods:**

Serum levels of semaphorin 3A, 3F, 4A, 4D, and 7A were compared between 41 patients with MG and 39 healthy controls. Patients were grouped according to the Myasthenia Gravis Activities of Daily Living scale, and differences in semaphorin levels between groups were analyzed.

**Result:**

Semaphorin 4A levels were significantly lower, whereas semaphorin 7A levels were higher in patients with MG than in controls. However, no significant correlation was found between the disease stage and semaphorin levels.

**Conclusion:**

Our findings suggest that the levels of semaphorin 4A and 7A may not only support the diagnosis of MG and aid in differential diagnosis but also shed light on the development of future therapeutic protocols targeting semaphorin proteins and receptors in other autoimmune and inflammatory diseases.

## Graphical abstract

Serum alterations of immune‐related semaphorins in myasthenia gravis. Patients exhibited decreased semaphorin 4A and increased semaphorin 7A levels compared with healthy controls. These changes highlight disturbed immune regulation in myasthenia gravis and support the involvement of semaphorin‐mediated pathways in autoimmune neuromuscular junction disorders.

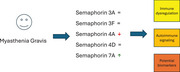

## 1 Introduction

Myasthenia gravis (MG) is an autoimmune disorder characterized by the presence of antibodies against nicotinic acetylcholine receptors (AChR) on the postsynaptic membrane at the neuromuscular junction (NMJ) of the skeletal muscles (Deymeer 2020). Since the identification of the first antibody in 1976, our understanding of MG pathophysiology and immunology has significantly expanded (Lindstrom et al. 1976).

Pathogenic anti‐AChR antibodies are high‐affinity immunoglobulin G (IgG) molecules. Activated CD4+ T cells are required for synthesis, interaction, and stimulation of B cells. Therefore, thymectomy to eliminate AChR‐specific CD4+ T cells may help alleviate symptoms in patients with MG. Similarly, anti‐CD4+ antibody treatment has also demonstrated therapeutic effects (Lewis 2013). In cases in which anti‐AChR antibodies are undetectable, approximately 40% of patients with MG may present with muscle‐specific kinase (MuSK) antibodies. Anti‐MuSK antibodies disrupt the organization of AChR in the NMJ, thereby reducing receptor functionality and efficacy (Rodolico et al. 2020). Diagnostic studies on MG have largely focused on detecting antibodies, with approximately 80%–85% of patients with generalized MG showing positivity for AChR antibodies and 5%–7% for MuSK antibodies. More recently, other antibodies have been detected in seronegative patients with MG, who were previously considered to lack detectable autoantibodies. The less commonly defined antibodies in MG include those targeting MuSK, low‐density lipoprotein receptor‐related protein 4 (Lrp4), and agrin (Dresser et al. 2021). Despite a small percentage (5%–7%) of patients with generalized MG being seronegative, ocular MG presents with 40%–50% seronegativity (Engel 2013; Öge et al. 2021).

This robust immune response observed in patients with MG has prompted researchers to explore the molecules involved in immune regulation and search for biomarkers that could measure disease onset and activity. Semaphorins are extracellular signaling proteins that are essential for the development and maintenance of various organs and tissues (Alto and Terman 2017). Initially characterized as guide molecules in neuronal development, semaphorins have since been implicated in autoimmune diseases, neurodegenerative conditions, musculoskeletal disorders, cardiovascular diseases, and cancers (Fard and Tamagnone 2021; Urhan et al. 2023). Over 20 semaphorins have been identified and categorized into five classes (Semaphorins 3–7) based on their structural characteristics (Neufeld and Kessler 2008).

Since their discovery, numerous studies have investigated interactions between semaphorins and neuropilin receptors. These interactions offer strong evidence for the involvement of semaphorins in the pathophysiology of MG, a disease with thymic involvement. Neuropilins, especially neuropilin 1 (NRP1), are key receptors for semaphorin 3. Semaphorins bind to neuropilin receptors (NRP1 or NRP2), forming complexes with the Plexin A family and transmitting signals to cells (Christie et al. 2021). NRP1 is highly expressed in CD4+ T helper (T‐h) cells, including regulatory T cells (T‐regs), which play crucial roles in immune modulation. In addition, NRP1 has been highlighted as a useful marker for distinguishing between thymus‐derived and peripheral adaptive T‐reg cells (Weiss et al. 2012; Chen et al. 2022).

Studies have confirmed the role of semaphorins in the neuromuscular (NM) area, and several studies have demonstrated their activity in the central nervous system (CNS). For example, semaphorin 3A protects neurons from attack by inducing apoptosis in activated microglial cells and plays a neuroprotective role in cortical neurons (Majed et al. 2006). Semaphorin 3F, a Class 3 semaphorin, is located at the 3p21.3 region of Chromosome 3 and plays a role in inhibiting angiogenesis, lymphangiogenesis, and invasion in tumor tissues (Futamura et al. 2007; Doci et al. 2015). Semaphorin 4A, an immune‐related semaphoring, is crucial for the immune responses between dendritic cells (DCs) and T cells. DC‐derived semaphorin 4A prepares antigen‐specific T cells, while semaphorin 4A is critical for cell differentiation of T‐h cells (Kumanogoh et al. 2002). Semaphorin 4D, another immune semaphorin, is expressed on the surface of various immune cells, including T cells, B cells, neutrophils, macrophages, and platelets (Huang et al. 2020). Semaphorin 7A acts as a chemoattractant for monocytes/macrophages and stimulates the release of proinflammatory cytokines. It has been shown that semaphorin 7A expression increases in inflammatory cells infiltrating the CNS and in circulating immune cells following experimental autoimmune encephalomyelitis (EAE) induction (Gutiérrez‐Franco et al. 2016).

For over 30 years, the semaphorin family, known for promoting axonal growth, has been extensively studied in autoimmune diseases, including MG, in which multiple immune mechanisms and autoantibodies besides anti‐AChR and anti‐MuSK are involved. In this study, we examined the relationship between the immune‐regulatory semaphorins 3A, 3F, 4A, 4D, and 7A and MG, a disease involving diverse immune mechanisms. Additionally, we explored the potential link between semaphorins and thymomas in patients with MG because some semaphorins are known to interact with tumor suppressor genes.

Although most studies have focused on experimental models investigating the role of semaphorin–neuropilin–plexin signaling pathways in the immunopathogenesis of MG, there is a lack of human serum‐based studies. Therefore, our findings provide novel evidence supporting a potential role of immune‐regulatory semaphorins in the immunopathogenesis of MG and may guide future studies on neuroinflammation and neuroimmunology.

## 2 Materials and Methods

Ethical approval of the study was received from Sakarya University Faculty of Medicine Ethics Committee with the number E‐71522473‐050.01.04‐233251‐102. The study group included 41 patients aged 18–65 years selected from a total of 98 patients with MG, followed up at the NM disease outpatient clinic.

Patients with any known inflammatory diseases (such as rheumatologic diseases, infections, and multiple sclerosis‐like neuroimmunological diseases), autoimmune diseases, or malignancies other than thymoma were excluded from the study. All patients included in the study were positive for AChR antibodies. Patients who were seronegative or positive for anti‐MuSK antibodies were excluded from the study. Ten patients who received intravenous immunoglobulin (IVIg) and five patients who underwent plasmapheresis were not included in the study during the active treatment period. Blood samples for the study were obtained at least 1 month after completion of IVIg or plasmapheresis.

The control group consisted of 39 healthy individuals aged 18–65 years with no history of chronic diseases, infections, autoimmune diseases, malignancies, or regular medication use.

All participants were provided with information about the study and written and verbal informed consent was obtained from both the patients and control subjects.

### 2.1 Assessment of Patients

Demographic characteristics, routine blood parameters, disease features, and clinical findings of the patients with MG were recorded on a patient information form. Serum samples from both the patient and control groups were stored at −80°C until semaphorin levels were measured.

### 2.2 Clinical Assessment

The clinical severity of MG was assessed using the Myasthenia Gravis Activities of Daily Living (MG‐ADL) scale (Muppidi et al. 2011).

### 2.3 Measurement of Semaphorin Levels

Serum semaphorin levels in both patient and control groups were measured at the Sakarya University Medical Faculty Microbiology Laboratory by specialized personnel. Commercially available ELISA kits were employed for in vitro quantitative determination of human semaphorin concentrations in serum, plasma, and other biological fluids. The kits primarily follow the Sandwich‐ELISA principle. The micro‐ELISA plate in the kit was pre‐coated with an antibody specific to the particular semaphorin subgroup being analyzed (semaphorin 3A, 3F, 4A, 4D, or 7A). Each subgroup has a separate antibody to ensure specificity. Samples were added to the micro‐ELISA plate wells and incubated with specific antibodies. A biotinylated detection antibody specific for human semaphorin and an avidin‐horseradish peroxidase (HRP) conjugate were sequentially added to each well and incubated. The free components were washed, and a substrate solution was added to each well. Only the wells containing human semaphorin, the biotinylated detection antibody, and avidin‐HRP conjugate produced a blue color, indicating the presence of the target semaphorin. The enzyme‐substrate reaction was halted with a stop solution, turning the color yellow. Optical density (OD) was measured spectrophotometrically at a wavelength of 450 ± 2 nm. The OD value is directly proportional to the concentration of human semaphorin. The concentration of human semaphorin in the samples was calculated by comparing the OD values with a standard curve.

### 2.4 Statistical Analysis

Statistical analyses were performed using SPSS version 21 software. The normality of variables was assessed visually (histograms and probability plots) and analytically (Kolmogorov–Smirnov and Shapiro–Wilk tests), along with examining skewness and kurtosis values, which were maintained within the ± 1.5 range. Descriptive statistics were presented as mean and standard deviation for variables with normal distribution and as median and interquartile ranges for non‐normally distributed variables (frequency tables were used for categorical variables). The frequencies of the categorical variables across groups were presented in cross‐tabulations. Differences between the groups in terms of these frequencies were assessed using Pearson's chi‐square test. For the comparison of non‐normally distributed variables between groups, the Mann–Whitney *U* test was applied. The Spearman's correlation test was used to examine the relationships between non‐normally distributed variables. Statistical significance was set at *p* < 0.05.

## 3 Results

The sex and age distributions of the 41 patients and 39 control individuals included in our study were similar in both groups (Table 1). Serum semaphorin 4A levels were significantly lower in the patient group than in the control group (*p* = 0.000). However, serum semaphorin 7A levels were significantly higher in the patient group than in the control group (*p* = 0.000). There were no significant differences in the serum semaphorin 3A, 3F, and 4D levels between the patient and control groups (*p* = 0.624, 0.862, and 0.195, respectively) (Table 2). No statistical relationship was found between serum semaphorin 3A, 3F, 4A, 4D, and 7A levels and MG‐ADL stage (*r* = 0.018, 0.211, 0.116, 0.206, −0.003; *p* = 0.911, 0.186, 0.471, 0.197, and 0.984, respectively) (Table 3).

**TABLE 1:** The sex and age distributions of the groups.

	F	M	TV	*p*	*n*	MEAN	SD	MIN	MAX	MED	IQR	TV	*p*
PATIENT	22 (53.7%)	19 (46.3%)	0.000	0.987	41	61.46	13.7	30	88	63	21	−1.137	0.256
CONTROL	21 (53.8%)	18 (46.2%)			39	64.56	9.5	51	80	64	17		

Abbreviations: F, female; M, male; TV, test value; SD, standard deviation; Min, minimum; Max, maximum; Med, median; IQR, interquartile range.

**TABLE 2:** Comparison of average semaphorin 3A, 3F, 4A, 4D, and 7A levels of the patient and control groups.

ng/mL	Group	*n*	Mean	SD	Min	Max	Med	IQR	Test statistics	*p*
3A	Patient	41	1.33	1.03	0.1	6.68	1.1	0.73	−0.490	0.624
	Control	39	2.37	2.35	0.1	8.44	3.05	3.96		
3F	Patient	41	11.17	5.46	6.56	25.00	8.89	4.46	−0.173	0.862
	Control	39	9.83	3.46	5.70	25.00	9.00	3.07		
4A	Patient	41	10.62	6.47	3.79	25.00	8.47	4.58	−4.626	**0.000**
	Control	39	14.86	3.17	9.43	25.00	14.80	4.40		
4D	Patient	41	2.24	1.14	1.50	9.10	1.99	0.47	−1.296	0.195
	Control	39	3.01	4.16	1.50	26.40	1.89	1.03		
7A	Patient	41	4.04	1.04	1.54	7.46	3.95	1.20	−7.292	**0.000**
	Control	39	1.95	0.55	0.85	3.48	1.90	0.85		

*Note*: Mann–Whitney *U* Test, *p* < 0.05 is significant.

Abbreviations: IQR: interquartile range, Max: maximum, Med: median, Min: minimum, SD: standard deviation.

**TABLE 3:** Relationship between serum semaphorin 3A, 3F, 4A, 4D, and 7A levels and patients' MG‐ADL scores.

Serum level (ng/mL)		MG‐ADL score
3A	*r*	0.018
	*p*	0.911
	*n*	41
3F	*r*	0.211
	*p*	0.186
	*n*	41
4A	*r*	0.116
	*p*	0.471
	*n*	41
4D	*r*	0.206
	*p*	0.197
	*n*	41
7A	*r*	−0.003
	*p*	0.984
	*n*	41

*Note*: *r*. Spearman correlation test; *p* < 0.05 is significant.

Abbreviation: MG‐ADL = Myasthenia Graves‐ Activities of Daily Living.

In the MG group, 10 of 41 patients underwent thymectomy. Among the patients who underwent thymectomy, four were reported to have thymoma, five had thymic hyperplasia, and one had normal thymic tissue. There were no significant differences in serum semaphorin 3A, 3F, 4A, 4D, and 7A levels between patients who did and did not undergo thymectomy (*p* = 0.286, 0.170, 0.137, 0.393, and 0.671, respectively) (Table 4).

**TABLE 4:** Comparison of average semaphorin 3A, 3F, 4A, 4D, and 7A levels in the patient group with and without thymectomy.

ng/mL	Thymectomy	*n*	Mean	SD	Min	Max	Med	IQR	Test statistics	*p*
3A	−	31	1.27	1.11	0.1	6.68	1.27	0.72	−1.093	0.286
	+	10	1.52	0.69	0.82	2.63	1.35	1.2		
3F	−	31	10.36	4.97	6.56	25.00	8.82	1.86	−1.382	0.170
	+	10	13.68	6.39	7.60	25.00	11.68	12.13		
4A	−	31	9.41	5.37	3.79	25.00	8.11	3.16	−1.489	0.137
	+	10	14.35	8.32	5.24	25.00	12.90	19.03		
4D	−	31	2.29	1.30	1.50	9.10	2.00	0.36	−0.866	0.393
	+	10	2.08	0.39	1.76	2.68	1.86	0.80		
7A	−	31	4.02	0.94	2.71	7.46	3.95	0.82	−0.425	0.671
	+	10	4.13	1.35	1.54	6.51	4.11	1.68		

*Note*: Mann–Whitney *U* Test, *p* < 0.05 is significant.

Abbreviations: IQR: interquartile range, Max: maximum, Med: median, Min: minimum, SD: standard deviation.

Three patients in the MG group passed away. Among the deceased patients, two died due to infection‐related causes, and one patient died outside the hospital following abrupt discontinuation of all medications and the development of bulbar symptoms. Because the group sample sizes were insufficient, statistical comparisons could not be made between patients with and without exitus.

## 4 Discussion

### 4.1 Semaphorin 3A

Semaphorin 3A is known to be involved in central neuronal activity disturbances; however, our study found no significant changes in its levels in MG, an NMJ disorder influenced by thymus‐derived T‐cell dysfunction. One study showed that after peripheral nerve injury, the expression and secretion of semaphorin 3A in sensory Schwann cells (SC) were more significant than those in motor SCs, and the expression of its receptor, NRP1, in sensory neurons was higher than that in motor neurons. These findings suggest that semaphorin 3A secreted by SCs may play a selective role in the regeneration of sensory neurons through a preferential mechanism (Shen et al. 2023). The lack of sensory involvement in the MG may explain the similar semaphorin 3A levels observed between our study's MG and control groups. Birger et al. demonstrated that semaphorin 3A decreases the survival of human cortical neurons while promoting the survival of spinal motor neurons (Birger et al. 2018). This protein has been suggested to contribute to neuronal loss in the cortex of patients with amyotrophic lateral sclerosis (ALS) with increased semaphorin 3A signaling, which plays a role in axonal degeneration and motor neuron death in ALS (Korner et al. 2016). Given this information, it seems easier to associate semaphorin 3A with presynaptic disorders such as Lambert–Eaton myasthenic syndrome and botulismus. However, the absence of motor neurons in the postsynaptic area and the unaffected semaphorin 3A levels observed in our study seem consistent with this neuroanatomical situation. Semaphorin 3A has also been implicated in tissue repair following muscle injury and age‐related sarcopenia. These axon guidance molecules play crucial roles in tissue regeneration after muscle damage (Fard et al. 2024). Within the NM anatomical pathway, semaphorins and their neuropilin and plexin receptors aid axonal navigation, presynaptic–postsynaptic assembly, and muscle regeneration. However, to our knowledge, no prior studies have explored the relationship between semaphorin 3A and MG, a postsynaptic NM disorder, making our findings the first scientific evidence of semaphorin 3A levels in MG, providing a basis for future research in this area.

### 4.2 Semaphorin 3F

Studies have indicated that semaphorin 3F is involved in the CNS. In an experimental model, Beck et al. (2002) showed that both semaphorin 3A and 3F are strongly downregulated in cortical neurons in cerebral infarction and the surrounding areas. They also suggest that low levels of semaphorin 3 in neurons might support neuronal remodeling and recovery of neurological function following experimental cerebral ischemia (Beck et al. 2002). Although these findings provide strong evidence on the pyramidal tract, studies on the relationship between semaphorin 3F and NMJ are limited.

Semaphorin 3F signaling actively regulates neutrophil recruitment in injured peripheral tissues, resulting in the clearance of neutrophils and the resolution of inflammation (Plant et al. 2020). This study investigated whether semaphorin 3F, which is associated with inflammation, may be a prognostic factor in MG, an NM disease whose prognosis worsens with infections, and detected non‐significant data for semaphorin 3F in humans for the first time. Further studies on this protein are needed, which has not been previously shown to be associated with CNS or NM diseases in humans.

### 4.3 Semaphorin 4A

In a recent study, serum semaphorin 4A levels were found to be higher in patients with MG than in controls, and semaphorin 4A may reflect T‐cell activation in MG and be a potential biomarker for disease activation (Akiyuki et al. 2024). However, our study found the opposite result, with significantly lower serum semaphorin 4A levels in the MG group than in the control group.

Semaphorin 4A, a membrane‐bound protein expressed in immune cells, influences immune responses by binding to T‐cell surfaces. Experimental studies have shown that individuals with low levels of semaphorin 4A impair Th1 responses while promoting Th2‐dependent responses in nematode‐infected models. Semaphorin 4A has been suggested to enhance Th1‐driven diseases and suppress Th2‐mediated diseases (Misagh et al. 2020; Okuno et al. 2020). In the MG, CD4+ Th cells are crucial for producing antibodies involved in disease activation. Previous studies have emphasized the imbalance of Th1/Th2, numerical changes in T‐reg cells, and other factors, such as Th17, in the pathophysiology of MG (Wang et al. 2012).

Lower levels of semaphorin 4A in the MG group may be related to the varying clinical nature of the disease. We hypothesized that changes in the T‐cell balance due to disease activation, remission, or intervening infections might lead to varying semaphorin 4A levels. Further studies examining semaphorin levels in patients undergoing steroid treatment, particularly comparing subgroups in the remission and non‐remission states, will shed more light on this issue.

### 4.4 Semaphorin 4D

Semaphorin 4D is associated with various inflammatory conditions, such as rheumatoid arthritis, vasculitis, and coronary artery stenosis (Huang et al. 2020). This protein is expressed in endothelial cells and monocytes, plays a role in endothelium–monocyte interactions, and influences cytokine production. Under hypoxic conditions, its expression increases in microvascular endothelial cells, and its overexpression has been shown to significantly enhance angiogenesis and inhibit axon myelination (Zhang et al. 2014). Semaphorin 4D, produced in oligodendrocytes, serves as an axon‐guidance molecule and plays a critical role in regulating axonal growth, with increased levels observed in CNS lesions and spinal cord injuries (Misagh et al. 2020). In another study, mice with semaphorin 4D deficiency were resistant to EAE, an animal model of multiple sclerosis (Okuno et al. 2010). Semaphorin 4D has also been associated with neurodegenerative diseases; in experimental models, it has been observed that semaphorin 4D upregulates neurons during disease progression in Huntington's and Alzheimer's disease (Evans et al. 2022).

Our study found no significant relationship between semaphorin 4D levels and MG. Based on these findings, semaphorin 4D may be a strong biomarker for inflammatory, autoimmune, demyelinating, and neurodegenerative diseases of the CNS. Further studies are required to establish its role in NM disorders.

### 4.5 Semaphorin 7A

Semaphorin 7A is associated with several autoimmune and inflammation‐related diseases, including tumors. Our study found that serum levels of semaphorin 7A were significantly higher in the MG patient group than in controls. A study in patients with rheumatoid arthritis demonstrated that the upregulation of semaphorin 7A in both the serum and synovial fluid was correlated with disease activity. Semaphorin 7A contributes to inflammation and disease progression in rheumatoid arthritis, an autoimmune and inflammatory disorder (Xie and Wang 2017).

In a more recent study, semaphorin 7A levels were measured in serum samples from patients with Kawasaki disease via ELISA. The results showed significantly higher levels of semaphorin 7A, which contributes to disease pathogenesis by increasing endothelial permeability and inflammatory responses (Huang et al. 2024).

Furthermore, it is believed that semaphorin 7A plays a role in peripheral immunity and inflammation in multiple sclerosis. High levels of semaphorin 7A have been observed near inflammatory plaques in the CNS in experimental models (Carulli et al. 2021). Both experimental studies and human serum samples suggest that semaphorin 7A could be an important biomarker and prognostic factor for autoimmune inflammatory conditions.

There are no previous studies on human serum levels of semaphorin 7A in autoimmune NM disorders. To our knowledge, this is the first study to demonstrate elevated serum semaphorin 7A levels in patients with MG, suggesting a possible association with immune dysregulation in this disease.

Although these findings indicate that semaphorin 7A reflects disease‐related immune activity at the group level, the substantial overlap between patient and control values limits its utility as a stand‐alone diagnostic biomarker. Rather, semaphorin 7A may represent a complementary indicator of immune activation and warrants further investigation in larger, longitudinal cohorts.

Semaphorin 3 is a secreted protein, whereas semaphorins 4–7 are membrane‐bound proteins (Misagh et al. 2020). It is therefore reasonable to expect that semaphorin 4 and 7 levels might change in parallel during autoimmune activity. However, in our study, semaphorin 4A levels were decreased while semaphorin 7A levels were increased. This discrepancy may reflect disease heterogeneity, treatment effects, or dynamic immune regulation. Although patients receiving IVIg or plasmapheresis were excluded, the widespread use of corticosteroids in our cohort may have influenced serum semaphorin levels. Future studies including treated and untreated subgroups will be necessary to clarify these effects.

We examined the potential use of semaphorins as prognostic biomarkers by comparing their levels with the MG‐ADL scores of patients. No significant correlation was observed between the semaphorin levels and disease severity. These results suggest that semaphorins 3A, 3F, 4A, 4D, and 7A are not useful prognostic biomarkers. However, this area remains uncertain, and further studies with larger patient cohorts and treatment‐based analyses are required.

Furthermore, the thymus plays a significant role in triggering inflammation and autoimmunity. No significant differences were found in semaphorin levels between patients who underwent thymectomy and those who had not. Semaphorins are frequently discussed for their tumor‐suppressive properties, and their expression has been observed in tumor tissues. This suggests that the relationship between semaphorins, thymomas, and thymic hyperplasia may stem from their connection to tumor tissues and inflammatory processes. The limited sample size and number of patients with thymomas may have contributed to these statistically insignificant results. Future studies on semaphorins involving larger patient cohorts, including pre‐ and post‐thymectomy cases including those with thymoma and thymic hyperplasia, will provide valuable contributions to the literature.

Our study has some limitations. It lacked a sufficient sample size to determine the role of semaphorins as biomarkers of mortality. Other limitations include the small number of patients and the fact that patients' treatment protocols were not included in the study.

## Author Contributions

**Dilcan Kotan**: conceptualization, methodology, data curation, formal analysis, writing – original draft, writing – review and editing. **Esen Çiçekli**: conceptualization, writing – original draft, methodology, validation, visualization, writing – review and editing, data curation, resources. **Özlem Aydemir**: data curation, formal analysis, investigation.

## Funding

The study was funded by Türkiye Sağlık Enstitüleri Başkanlığı and Türkiye Bilimsel ve Teknolojik Araştırma Kurumu.

## Conflicts of Interest

The authors declare no conflicts of interest.

## Data Availability

The data that support the findings of this study are available on request from the corresponding author. The data are not publicly available due to privacy or ethical restrictions.

## 1

The levels of inflammatory semaphorins, particularly semaphorins 4A and 7A, were significantly different between patients with MG and controls. These findings suggest that semaphorin alterations reflect disease‐related immune activity at the group level. However, the substantial overlap between groups limits their utility as stand‐alone diagnostic markers. Rather, semaphorins may provide insight into disease mechanisms and represent potential targets for future mechanistic and translational research. These observations may also be relevant to other autoimmune and inflammatory disorders.
